# Glioma-associated oncogene homolog 1 in breast invasive carcinoma: a comprehensive bioinformatic analysis and experimental validation

**DOI:** 10.3389/fcell.2024.1478478

**Published:** 2024-10-17

**Authors:** Teng Qi, Yujie Hu, Junhao Wan, Bo Zhao, Jinsuo Xiao, Jie Liu, Ye Cheng, He Wu, Yonggang Lv, Fuqing Ji

**Affiliations:** ^1^ School of Medicine, Northwest University, Xi’an, Shaanxi, China; ^2^ Xi’an NO.3 Hospital, The Affiliated Hospital of Northwest University, Xi’an, Shaanxi, China

**Keywords:** glioma-associated oncogene homolog 1, breast invasive carcinoma, immune infiltration, prognostic, tumor immune microenvironment

## Abstract

**Background:**

Breast cancer, despite significant advancements in treatment, remains a major cause of cancer-related deaths among women. Immunotherapy, an emerging therapeutic strategy, offers promise for better outcomes, particularly through the modulation of immune functions. Glioma-Associated Oncogene Homolog 1 (GLI1), a transcription factor implicated in cancer biology, has shown varying roles in different cancers. However, its immunoregulatory functions in breast invasive carcinoma (BRCA) remain elusive. The current study aimed to unravel the expression patterns and immune-regulatory roles of GLI1 in BRCA.

**Methods:**

Utilizing multiple bioinformatic platforms (TIMER2.0, GEPIA2, and R packages) based on The Cancer Genome Atlas (TCGA) and/or Genotype-Tissue Expression (GTEx) databases, we analyzed the expression of GLI1 in BRCA and its pan-cancer expression profiles. We further validated these findings by conducting qPCR and immunohistochemical staining on clinical BRCA samples. Kaplan-Meier analysis and Cox proportional hazards regression were performed to assess the prognostic value of GLI1. Additionally, the association between GLI1 expression and immune infiltration within the tumor immune microenvironment (TMIE) was examined.

**Results:**

The findings reveal dysregulated expression of GLI1 in numerous cancers, with a significant decrease observed in BRCA. High GLI1 expression indicated better survival outcomes and was correlated with the age and stage of BRCA patients. GLI1 was involved in immune status, as evidenced by its strong correlations with immune and stromal scores and the infiltration levels of multiple immune cells. Meanwhile, GLI1 was co-expressed with multiple immune-related genes, and high GLI1 expression was associated with the activation of immune-related pathways, such as binding to proteasome and mismatch repair and retinol metabolism signaling pathways. Additionally, the differential expression of GLI1 may be related to the effect of immunotherapy on CTLA-4, PD-1, and other signals, and can effectively predict the immune efficacy.

**Conclusion:**

Our study underscores the critical role of GLI1 in BRCA, both as a potential tumor suppressor and an immune regulator. The association between GLI1 expression and favorable prognosis suggests its potential as a prognostic biomarker and immunotherapeutic target in BRCA.

## 1 Introduction

Breast cancer presents high heterogeneity in histology, genetics, and prognosis (Prat and Perou, 2011) ranking as the second leading cause of cancer-related deaths in women, following lung cancer (DeSantis et al., 2011). It is commonly categorized into three major subtypes based on the expressions of estrogen receptor (ER), progesterone receptor (PR), and human epidermal growth factor 2 (HER2/ERBB2): luminal type (hormone receptor positive/ERBB2 negative, accounting for 70% of patients), ERBB2 positive (15%–20%), and triple-negative (TNBC, representing 15% of cases) (Sørlie et al., 2001; Hui and Angers, 2011). The primary treatment modalities for breast cancer include surgery, conventional chemotherapy, and radiotherapy (often involving targeted drugs), but many patients encounter resistance, relapse, and secondary metastasis (Loibl et al., 2021). Despite advancements in multimodal therapy, early-stage breast cancer is considered curable in 70%–80% of cases, while managing advanced metastatic breast cancer remains a formidable challenge (Riggio et al., 2021). Immunotherapy has emerged as a promising approach in cancer treatment, showing efficacy across various cancer types (Zhang and Zhang, 2020; Chow et al., 2022). Although some immunotherapies, such as pembrolizumab (Nanda et al., 2020), and opdivo combined with ipilimumab, have demonstrated prolonged survival in breast cancer, a comprehensive understanding of immune infiltrates within the tumor immune microenvironment (TMIE) of BRCA is crucial for the development of more precise immunotherapeutic strategies (Adams et al., 2022).

Glioma-Associated Oncogene Homolog 1 (GLI1) was first identified as an amplified gene in human malignant glioma (Kinzler et al., 1987), and subsequently recognized as a member of the Kruppel family of zinc finger-containing transcription factors (Kinzler et al., 1988; Kinzler and Vogelstein, 1990). GLI1 is recognized as the nuclear mediator of the Shh signaling pathway, which governs diverse aspects of early central nervous system development (Dahmane et al., 2001), including potential involvement in floor cell and ventral neuron differentiation in the neural tube, proliferation of granule cell precursors in the cerebellum, and growth of the dorsal brain (Echelard et al., 1993; Roelink et al., 1994; Dahmane et al., 2001). In previous studies, it has been widely postulated that the GLI1 protein family potentially exerts a significant impact on tumor progression and metastasis (Zhu and Lo, 2010). In addition, accumulating evidence has shown that GLI1 plays a crucial role in regulating numerous cellular processes relevant to cancer, including cancer stemness, epithelial-mesenchymal transition, proliferation, cell cycle regulation, cell survival, programmed cell death, and reduced cell viability (Sun et al., 2014; Xu et al., 2014). Consequently, GLI1 contributes to the progression of multiple cancer types, including colorectal cancer, liver cancer, stomach cancer, pancreatic cancer, lung cancer, gallbladder cancer, prostate cancer, ovarian cancer, and glioblastoma (Akiyoshi et al., 2006; Olive et al., 2009; Kandala and Srivastava, 2012; Fernandez-Zapico, 2013; Gao et al., 2013; Li et al., 2016; Gu et al., 2021; Ichimiya et al., 2021; Quan et al., 2022; Wu et al., 2022). In addition, GLI1 appears to regulate tumor progression and metastasis by mediating components of TMIE. For example, GLI1 is co-ectopically removed with pSTAT3 by chondroitin-6-sulfate (C-6-S) to activate the JAK/STAT3 and Hedgehog pathways to induce immune rejection (Wu et al., 2022), through the activity of the aPKC-ι/GLI1 pathway (Tian et al., 2018). Promote GLI1 nuclear localization and binding to target gene promoter, promote gallbladder cancer metastasis and macrophage recruitment. These properties make GLI1 a promising target for cancer treatment. Nevertheless, the immunoinfiltration and tumor microenvironment of GLI1 in breast cancer have been poorly studied.

With the aim of comprehensively investigating the significance of GLI1 in breast invasive carcinoma (BRCA), this study was designed to address three key aspects. Firstly, it examined the expression of GLI1 in BRCA and its association with clinicopathological features and prognosis. Secondly, it explored the correlation between GLI1 expression and immune cell infiltration, as well as the levels of immune checkpoints within the TMIE, while analyzing the potential links between GLI1 expression and CTLA-4 and PD-1 immunotherapy. Lastly, it delved into the functional pathways related to GLI1 expression, aiming to uncover the underlying molecular mechanisms. The findings of this study will serve to provide valuable evidence regarding the role of GLI1 in breast cancer and its potential as both a prognostic biomarker and a therapeutic target.

## 2 Materials and methods

### 2.1 Data sources and differential analysis of GLI1 expression

Expression of GLI1 between tumor tissues and the corresponding normal tissues in pan-cancer were analyzed using the “Gene_DE Module” of TIMER2.0 (http://timer.cistrome.org/2022 accessed on 11 November 2023) (Li et al., 2020) and GEPIA (version 2, http://gepia.cancer-pku.cn/2022 accessed on 11 November 2023) (Tang et al., 2017). In addition, the transcriptome data of 33 pan-cancer types in the TCGA (https://tcga.xenahubs.net accessed on 11 November 2023, The abbreviations corresponding to the 33 cancer types and the analyzed sample sizes can be found in Supplementary Table 1) (Blum et al., 2018) were downloaded, and the differential expression of GLI1 was analyzed with the aid of the Wilcoxon test provided in the R package. Through these three ways, data were divided into high and low expression groups based on the median expression value of GLI1 expression. False discovery rate <0.05 indicated statistical significance.

Data from the TCGA online database, where the Transcripts Per Million (TPM) data did not contain zero values, allowing for the direct application of log2 (TPM). In contrast, incorporation data from the GTEx project, where the potential presence of zero values in the TPM data necessitated the use of log2 (TPM+1) to avoid mathematical errors in logarithmic calculations and ensure the validity of our statistical analysis.

Based on our analysis and the preliminary research directions of our department, we have decided to focus on a more in-depth investigation of GLI1 in invasive breast cancer. Gene expression profiles of BRCA and normal samples from the TCGA and GTEx (http://commonfund.nih.gov/GTEx/) (Lonsdale et al, 2013) databases were obtained for differential analysis of GLI1 expression between tumor and normal tissues using the Wilcoxon test. Statistical significance was determined at FDR <0.05. To mitigate potential batch effects arising from the use of different sequencing platforms and protocols, we analyzed the gene expression data from TCGA and GTEx databases separately using TIMER2.0 and GEPIA, both of which apply normalization procedures tailored to the respective data sources during preprocessing. TIMER2.0 integrates RNA sequencing data from various public databases, including TCGA and GTEx, and applies standardization methods such as Reads Per Kilobase Million (RPKM) or TPM to ensure comparability across samples. It also corrects for batch effects using algorithms like ComBat and addresses missing values through imputation techniques. Similarly, GEPIA2 utilizes TCGA and GTEx data, employing TPM standardization and conducting differential expression analyses with statistical methods such as t-tests and survival analyses. The expression distribution of GLI1 was visualized using the “ggpubr” R package, represented in the form of a boxplot.

### 2.2 Quantitative real-time PCR

Tissue samples were obtained from 5 patients diagnosed with breast invasive carcinoma, comprising both tumor and adjacent non-cancerous tissues. The samples were collected by the Breast Surgery Department of Xi’an NO.3 Hospital. The study protocol was approved by the Ethics Committee of Xi’an No. 3 Hospital, and informed consent was obtained from all patients for the collection of samples. All patients provided informed consent, and detailed diagnostic information is provided in Supplementary Table 2. The primers used in the experiments are listed in Table 1. Total RNA was extracted from these samples. Subsequently, cDNA was synthesized from the extracted RNA. Quantitative Real-Time PCR (qPCR) was then employed to assess GLI1 expression levels, with glyceraldehyde-3-phosphate dehydrogenase (GAPDH) serving as the internal reference gene for normalization. This approach ensures robust validation of GLI1 expression patterns in the context of breast invasive carcinoma. To analyze the differential expression of GLI1 in breast invasive carcinoma between cancer and normal groups, we conducted a two-sample *t*-test using the qPCR data. This statistical method compares the means of two groups to determine whether they are significantly different. In our analysis, we utilized the R language to perform the *t*-test, which provided us with a t-statistic, *p*-value, confidence interval for the difference in means, and sample estimates of the mean GLI1 expression levels for both groups.

**TABLE 1:** The primers used in the experiments.

Primer name	Primer sequence 5′-3′
Human-actin-F	CAG​ATG​TGG​ATC​AGC​AAG​CAG​GAG
Human-actin-R	GTC​AAG​AAA​GGG​TGT​AAC​GCA​ACT​AAG
Human-GLI1-F2	GCG​TGA​GCC​TGA​ATC​TGT​GTA​TG
Human-GLI1-R2	CGT​GGA​TGT​GCT​CGC​TGT​TG

### 2.3 Immunohistochemical staining

5 tumor specimens and 5 adjacent non-cancerous specimens were sectioned into 3.5 µm thick paraffin slices. Following dewaxing, rehydration, and antigen retrieval, the sections were incubated overnight at 4°C with anti-GLI1 antibody (SC-166907, 1:500, Santa Cruz, CA, United States). All slides were covered with poly-HRR goat antibody, followed by incubation with diaminobenzidine solution (3–5 min) and counterstaining with hematoxylin. Finally, the sections were observed under a fluorescence microscope.

### 2.4 GLI1 expression is associated with the prognosis of patients with BRCA

The clinicopathological and survival data of BRCA samples were obtained from the TCGA database. To assess the prognostic value of GLI1 in BRCA, all BRCA samples were categorized into high and low GLI1 expression groups based on their median expression values. Survival analysis, including overall survival (OS), disease-specific survival (DSS), progression-free interval (PFI), and disease-free interval (DFI), was performed using the R survival package, and the results were visualized as Kaplan-Meier curves with log-rank *p*-values. Additionally, the “limma” R package was used to conduct differential analysis of clinical information (including age, gender, tumor pathological stage, and tumor TNM stage) downloaded from the TCGA database, aiming to evaluate the association between GLI1 and clinical factors. The results were visualized through boxplots and heatmaps using the “ggpubr” and “ComplexHeatmap” R packages, respectively.

### 2.5 Immune infiltration in BRCA

The correlation between GLI1 and immune cell infiltration was assessed using the “Gene Module” of TIMER2.0 (Li et al., 2020). This module generated scatter plots displaying Spearman’s correlations between GLI1 expression and tumor purity, as well as the abundance of six immune cell types, including DCs, B cells, neutrophils, CD4^+^ T cells, macrophages, and CD8^+^ T cells. Additionally, stromal and immune scores were calculated using the R “ESTIMATE” package, with the sum of these scores representing the ESTIMATE score, which indirectly reflects tumor purity (Yoshihara et al., 2013). The infiltrating abundance of immune cells in BRCA tissues was estimated using the CIBERSORT algorithm, a computational method that quantifies fractions of 22 cell types from bulk tissue gene expression profiles. The xCELL algorithm was also employed to assess the immune cell infiltration in BRCA samples from TCGA database. xCELL is a computational method that estimates the abundance of various immune and stromal cell types in tumor tissues based on bulk gene expression profiles. Specifically, we utilized the Pearson correlation coefficient (Pearson R) to evaluate the correlation between the expression of GLI1, a key gene of interest, and the inferred abundances of various immune cell types. The Pearson correlation coefficient provides a measure of the linear dependence between two variables, allowing us to identify potential associations between GLI1 expression and immune cell infiltration patterns. The correlations between GLI1 expression, stromal and immune scores, and immune cell infiltration abundance were further analyzed using R packages such as “ggplot2,” “ggpubr” and “ggExtra.”

### 2.6 Co-expression analysis and immune checkpoint analysis in BRCA

Chemokine and other Immune genes are crucial in determining the composition of the tumor immune microenvironment. Co-expression analysis between GLI1 expression and the expression of immunosuppressive and immune activation genes, as well as MHC, chemokine, and chemokine receptor-related genes, was performed using the R “limma” package. Expression data of immune checkpoint genes were extracted, and their expression in two groups of BRCA samples stratified by high and low GLI1 expression was analyzed using the R “ggplot2” package. Furthermore, four groups of immune therapy scores of BRCA samples were downloaded from the TCIA database (https://tcia.at/ accessed on 11 November 2023). Differential analysis between the GLI1 expression groups and immune therapy scores was conducted using the R “limma” package, and the results were visualized through violin plots created with the R package “ggpubr.”

### 2.7 Gene set enrichment analysis (GSEA)

With the predefined GO and KEGG gene sets in GSEA website as an enrichment reference, GSEA was conducted to analyze the GO annotations terms and KEGG pathways that were significantly associated with GLI1 expression using the online visualization tool “sangerbox3.0”(http://www.sangerbox.com/ accessed on 11 November 2023).

### 2.8 Drug sensitivity analysis

We employed the Comprehensive Pancancer Analysis of Drug Sensitivity (CPADS) web platform (available at https://smuonco.shinyapps.io/CADSP/) to investigate the drug sensitivity profiles associated with the GLI1 gene in breast cancer (BRCA). The CPADS platform integrates data from multiple sources, including the Gene Expression Omnibus (GEO), TCGA, and Genomics of Drug Sensitivity in Cancer (GDSC) databases, allowing for comprehensive analysis of differential gene expression and drug response.

Specifically, we focused on the GLI1 gene to assess its influence on drug sensitivity in BRCA. Utilizing the GDSC and TCGA module within CPADS, we analyzed the IC50 values (half-maximal inhibitory concentration) of various anticancer drugs in BRCA samples with high and low expression levels of GLI1. The IC50 values represent the drug concentration required to inhibit 50% of cellular growth and are commonly used as a metric for drug sensitivity.

## 3 Results

### 3.1 Expression pattern of GLI1 in pan-cancer

**FIGURE 1:**
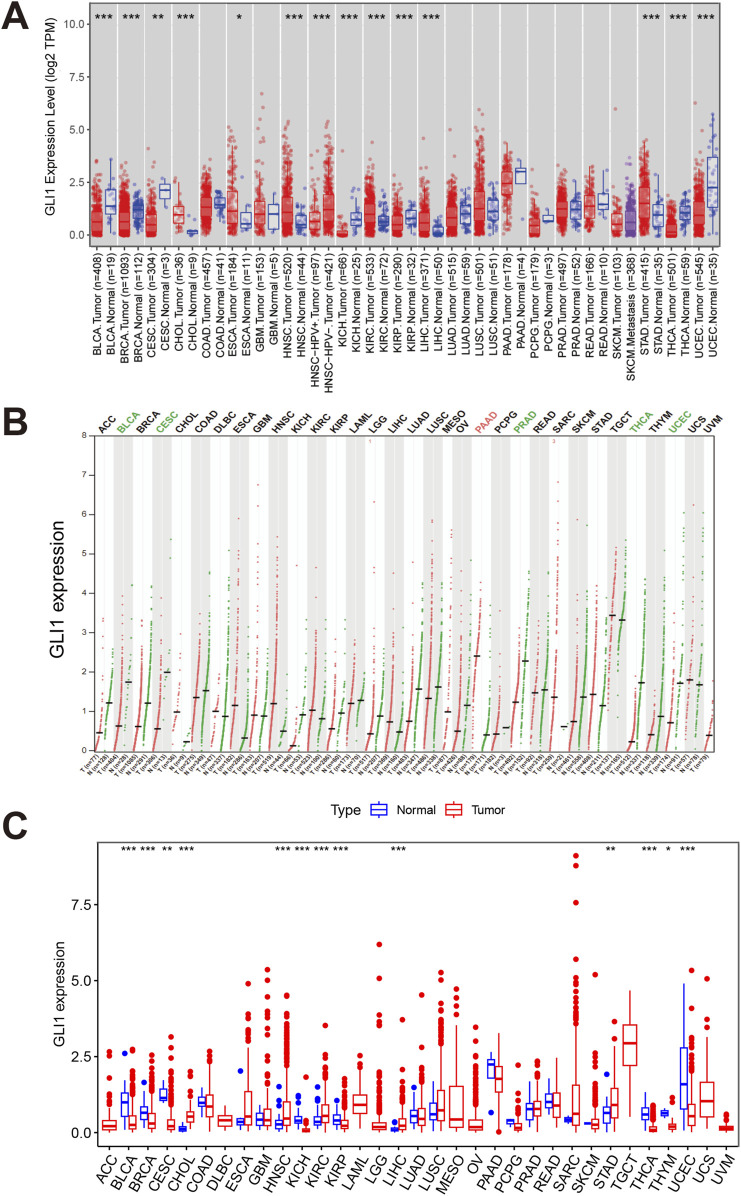
Expressions of GLI1 in pan-cancers. Expression levels of GLI1 in various cancers or tumor tissues and adjacent non-tumor tissues analyzed by TIMER2.0 **(A)**, GEPIA2 **(B)** and the R package **(C)** based on TCGA and/or GTEx databases. *p < 0.05, **p < 0.01, ***p < 0.001.

In order to evaluate the potential of GLI1 as a therapeutic target, we initially examined the expression pattern of GLI1 across various types of cancers, comparing tumor tissues with their corresponding normal tissues. Our analysis using TIMER2.0 revealed a significant reduction in GLI1 expression in tumor tissues compared to their matched normal controls in several cancer types, including BLCA, BRCA, KIRP, THCA, and UCEC. Conversely, GLI1 exhibited higher expression levels in CHOL, HNSC, KIRC, LIHC, and STAD compared to their matched controls (Figure 1A).

GEPIA, utilizing tumor tissues from TCGA and normal tissues from GTEx project, revealed the tumor-specific expression patterns of GLI1 (Figure 1B). Additionally, the R package was utilized to further analyze the expression levels of GLI1 across 33 cancer types in the TCGA database. The results were largely consistent with those obtained from TIMER2.0 (Figure 1C). GLI1 expression was found to be elevated in CHOL, HNSC, KIRC, LIHC, and STAD, while it was decreased in BLCA, BRCA, CESC, KIRP, THCA, THYM, and UCEC.

### 3.2 Expression pattern of GLI1 in breast invasive carcinoma

**FIGURE 2:**
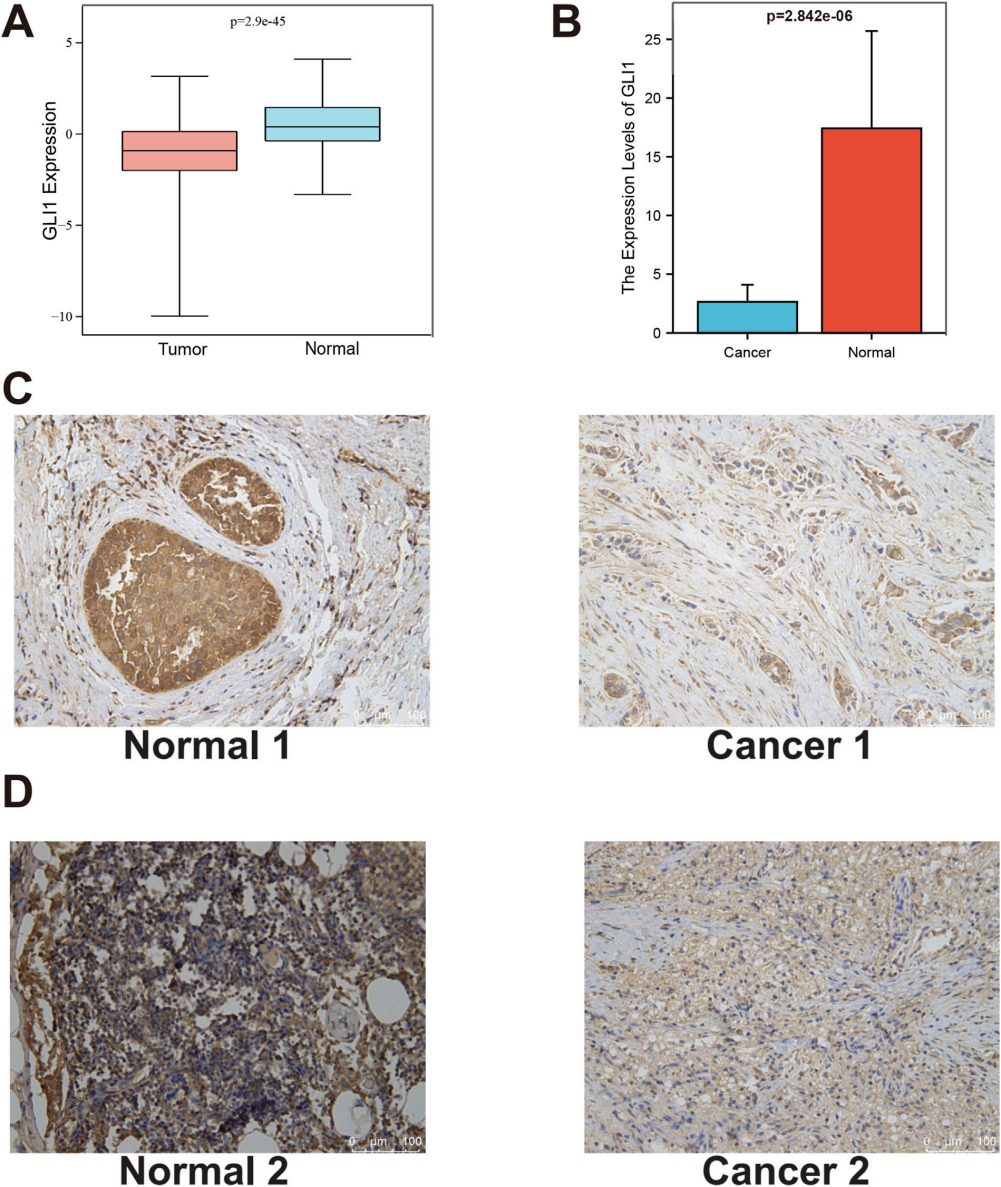
Expression pattern of GLI1 in BRCA. **(A)**, Differential expression of GLI1 between BRCA tissue and normal tissue samples in TCGA and GTEx; **(B)**, The qPCR results; **(C, D)**, Representative images and quantification of immunohistochemical staining for GLI1 in BRCA tissue and normal tissue for patient 1 and 2.

We conducted a comprehensive investigation into the expression distribution of GLI1 in BRCA. Analysis of sample data from TCGA and GTEx revealed a significant decrease in GLI1 expression in tumor samples compared to normal tissue samples (Figure 2A). Due to a high CT value and unexpected melting curve of actin amplification in a tumor sample, it was deemed unsuitable for qPCR experiments. Ultimately, we proceeded with qPCR on 4 tumor samples and 5 adjacent non-cancerous samples. Experimental results are presented in Figure 2B, indicating significantly higher expression in adjacent non-cancerous tissues compared to tumor tissues (*p* = 2.842e-06). Immunohistochemistry results, displayed in Figures 2C, D; Supplementary Figure 1, clearly show deeper staining intensity for GLI1 in adjacent non-cancerous tissues than in tumor tissues. GLI1 gene staining in breast cancer tissues appears lighter compared to adjacent tissues, suggesting lower GLI1 expression levels in breast cancer tissues, thus confirming the hypothesis from previous experimental data that gli1 gene functions as a tumor suppressor gene. This experiment was approved by the Ethics Committee of Xi’an NO.3 hospital. These findings collectively indicate a significant reduction in both mRNA and protein levels of GLI1 in BRCA tissue samples.

### 3.3 GLI1 expression is associated with prognosis of patients with BRCA

**FIGURE 3:**
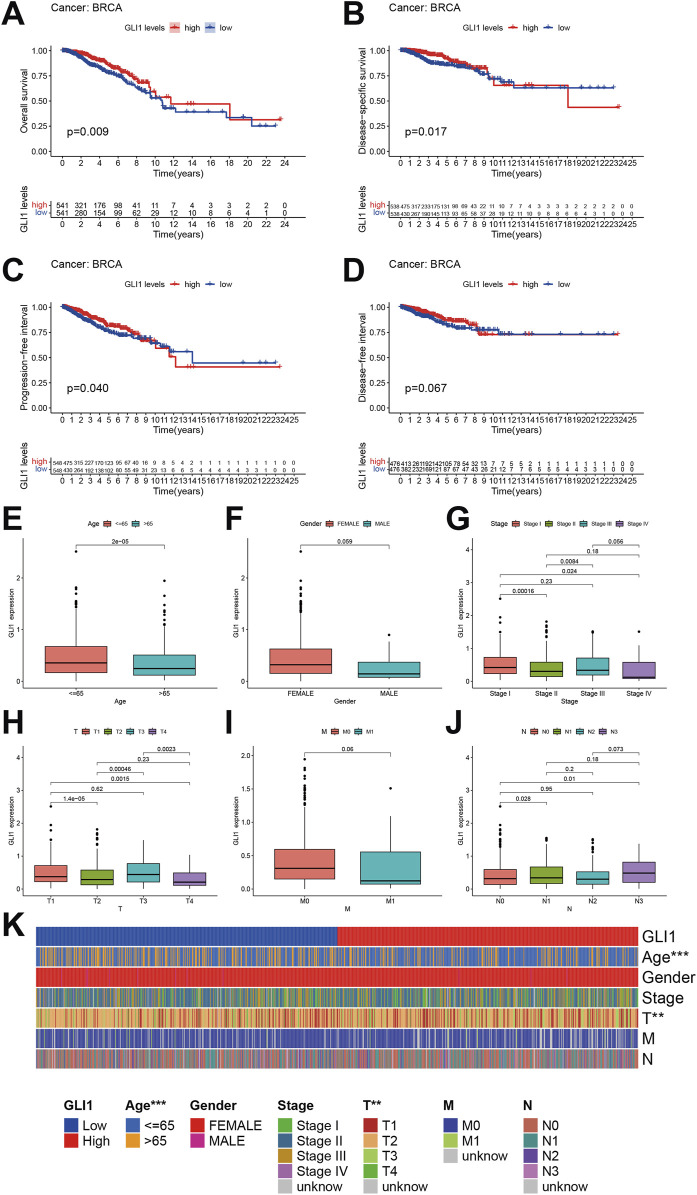
Prognostic value of GLI1 in BRCA. **(A–D)**, Survival curves showing the differences on overall survival **(A)**, disease-specific survival **(B)**, progression-free interval **(C)** and disease-free interval **(D)** between high and low GLI1 expression; **(E–J)**, Correlation box diagram of GLI1 expression difference and clinical factors; **(K)**, Heat map of correlation between GLI1 expression difference and clinical factors.

After adjusting for variables such as age, the prognostic significance of GLI1 in BRCA was assessed through survival analyses evaluating four key outcomes. Our findings revealed that BRCA patients with low GLI1 expression exhibited notably poorer overall survival (OS, *p* = 0.009), disease-specific survival (DSS, *p* = 0.017), and progression-free interval (PFI, *p* = 0.040) compared to those with high GLI1 expression (Figures 3A–C), highlighting the correlation between GLI1 expression and the survival outcomes of BRCA patients. However, no significant associations were found between GLI1 expression and the disease-free interval (DFI, *p* = 0.067) in BRCA patients (Figure 3D). It is important to highlight that the Kaplan-Meier plotter, based on disease-specific survival and progression-free interval, exhibits a crossing point around the 8th year. Notably, the survival outcome of the high-low expression group deviates from the aforementioned trend after the 8th year. This suggests that the effect of GLI1 expression on survival outcomes may be time-dependent, with GLI1 expression levels having a more pronounced influence in the earlier years post-diagnosis. These findings suggest a potential collaboration between GLI1 and other factors in influencing patient prognosis.

The correlation between the expression of GLI1 and various clinical factors, including age, sex, histopathological stage, and TNM stage (Figures 3E–J), was further investigated. Our analysis revealed that the expression of GLI1 was correlated with age, specific histopathological stages, and T stages (Figure 3K). Specifically, the expression of GLI1 was found to be significantly higher in patients aged ≤65 compared to those aged >65 (P = 2e-5). Furthermore, statistically significant differences in GLI1 expression were observed between stage I and stage II (*p* = 0.00016), stage I and stage IV (*p* = 0.024), as well as stage II and stage III (*p* = 0.0084) within the histopathologic staging. Similarly, significant variations in GLI1 expression were noted between T1 and T2 (*p* = 1.4e-05), T1 and T4 (*p* = 0.0015), T2 and T3 (*p* = 0.00046), as well as T3 and T4 (*p* = 0.0023) within the T stage of the tumor. However, no significant difference in GLI1 expression was observed between M0 (no distant metastasis) and M1 (presence of distant metastasis) stages in breast cancer (*p* = 0.06).

### 3.4 Association of GLI1 expression with immune infiltrates in BRCA

**FIGURE 4:**
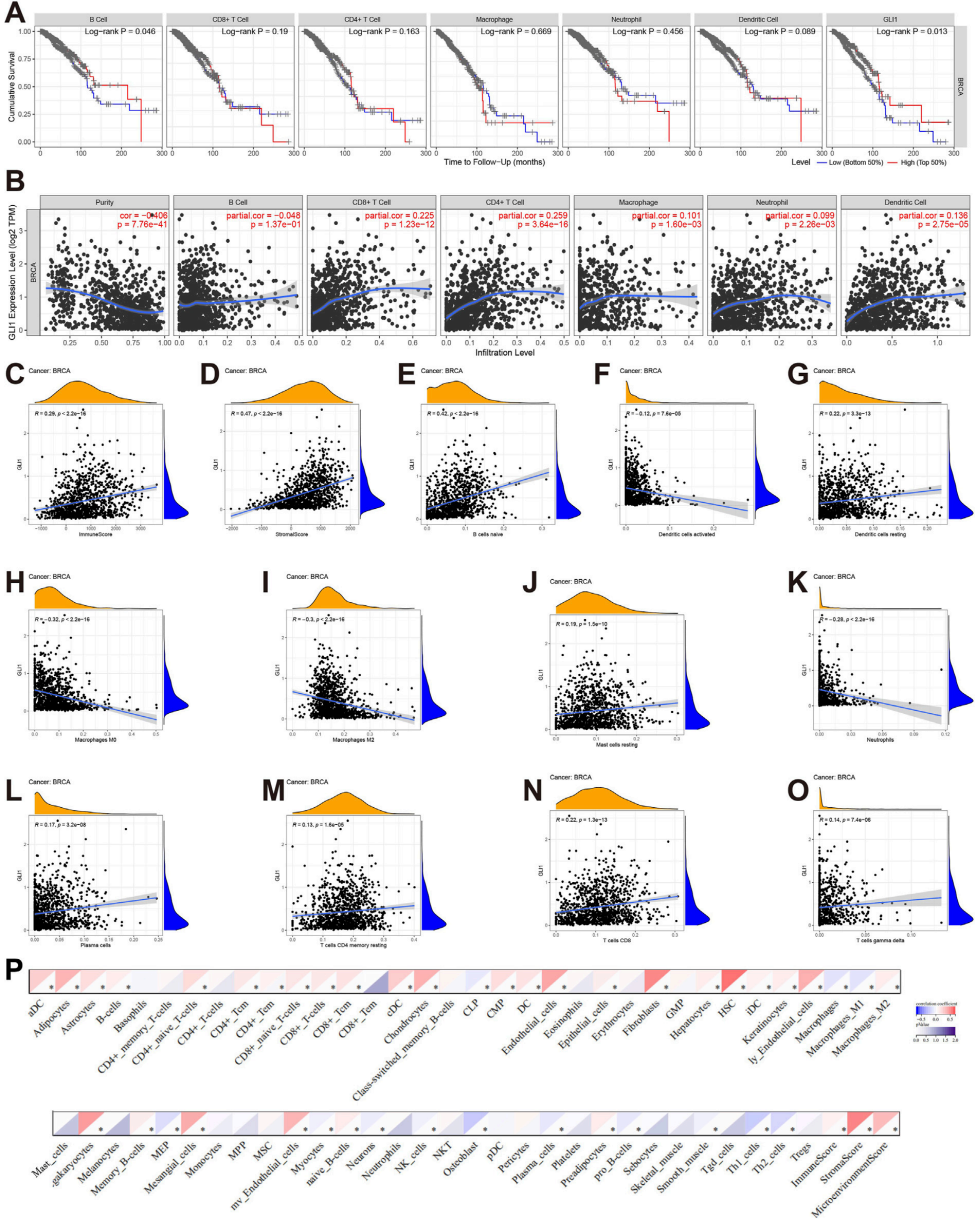
Associations of GLI1 with immune infiltration in BRCA. **(A)**, Cumulative survival of BRCA patients with high and low infiltrating levels of six immune cells in TIMER2.0; **(B)**, Correlations between GLI1 expression and infiltrating levels of six immune cells in TIMER2.0; **(C, D)**, Correlations of GLI1 expression with stromal **(C)** and immune **(D)** scores; **(E–O)**, Correlations between GLI1 expression and infiltrating levels of the immune cells by CIBERSORT; **(P)** Correlations between GLI1 expression and infiltrating levels of the immune cells by xCELL.

The involvement of GLI1 in the immune infiltrates within the TMIE of BRCA was further examined using various methods. The correlations between GLI1 expression and the infiltration levels of six main types of immune cells were explored using TIMER2.0. Patients with BRCA who exhibited low infiltration abundance of only B cells appeared to have a worse cumulative survival rate compared to those with high infiltration abundance (*p* = 0.046, Figure 4A). Notably, GLI1 expression exhibited significant positive correlations with the following immune cell types: DCs (r = 0.136, *p* = 2.75e-05), neutrophils (r = 0.099, *p* = 2.26e-03), CD4^+^ T cells (r = 0.259, *p* = 3.64e-16), CD8^+^ T cells (r = 0.225, *p* = 1.23e-12), and macrophages (r = 0.101, *p* = 1.60e-03). Conversely, GLI1 expression displayed a negative correlation with B cells (r = 0.183, *p* = 5.56e−5) and tumor purity (r = −0.406, *p* = 7.76e-14) in BRCA (Figure 4B). Furthermore, a significant positive correlation was observed between GLI1 expression and both stromal (r = 0.47, *p* < 2.2e−16) and immune (r = 0.42, *p* < 2.2e−16) scores in BRCA (Figures 4C, D). Additionally, using CIBERSORT, correlation analysis was performed to assess the relationship between GLI1 expression and the infiltration abundance of 11 immune cell types (Figures 4E–O). The results indicated that GLI1 expression positively correlated with naive B cells (r = 0.42, *p* < 2.2e-16), resting dendritic cells (r = 0.22, *p* = 3.3e-13), resting mast cells (r = 0.19, *p* = 1.5e-10), plasma cells (r = 0.17, *p* = 3.2e-08), resting memory CD4^+^T cells (r = 0.13, *p* = 1.6e-05), CD8^+^T cells (r = 0.22, *p* = 1.3e-13), and γδT cells (r = 0.14, *p* = 7.4e-06). Conversely, GLI1 expression exhibited negative correlations with activated dendritic cells (r = −0.12, *p* = 7.6e-05),macrophages M0 (r = −0.32, *p* < 2.2e−16), macrophages M2 (r = −0.3, *p* < 2.2e−16), and neutrophils (r = −0.28, *p* < 2.2e−16) in BRCA. Finally, the xCELL tool was employed to validate the infiltration of immune cells by GLI1, yielding results that were largely consistent (Figure 4P), thereby validating the robustness of the previously mentioned results.

### 3.5 Associations of GLI1 expression with immune status related genes and immunotherapy

**FIGURE 5:**
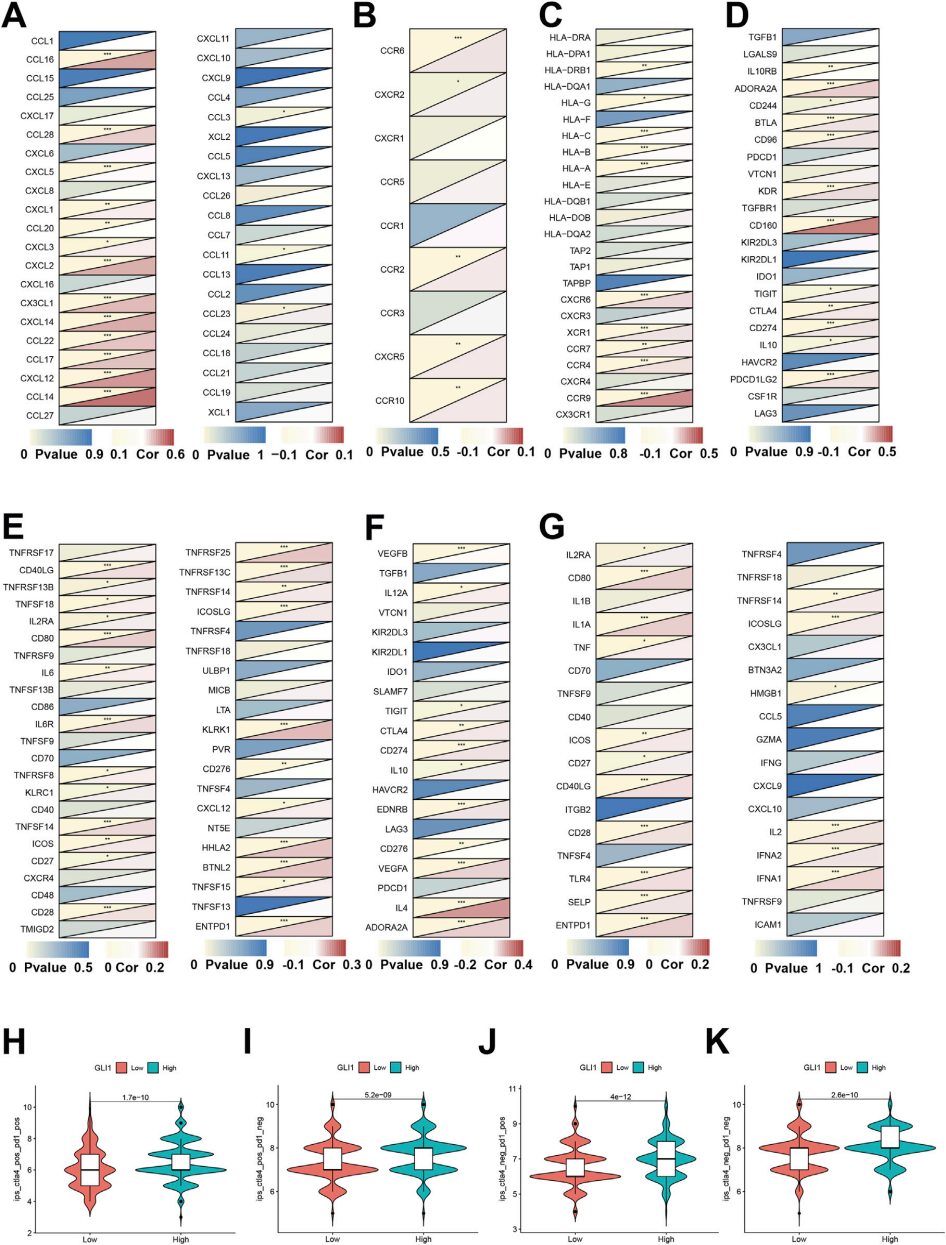
Associations of GLI1 expression with immune status-related genes and immunotherapy. Co-expression of GLI1 expression with chemokines **(A)**, receptors **(B)**, MHC **(C)**, immunoinhibitors **(D)**, immunostimulators **(E)**, immune checkpoint suppressor genes **(F)** and immune checkpoint-promoting genes **(G)**. Correlation analysis of gene expression and combined application of immunotherapy CTLA-4 and PD-1 **(H–K)** *p < 0.05, **p < 0.01, ***p < 0.001.

The expression of the GLI1 gene exhibited a strong positive correlation with the majority of chemokine genes, as illustrated in Figure 5A (e.g., CCL14, CXCL12, and CCL16). Conversely, the correlation analysis of receptor genes depicted minimal statistical significance, as demonstrated in Figure 5B. Upon examining the correlation with MHC genes, depicted in Figure 5C, we observed a significant positive correlation with certain genes (e.g., CCR9, etc.), and a notable negative correlation with another set of genes (e.g., HLA-A/B/C, HLA-DRB1, etc.). Analysis of the correlation between genes encoding immunoinhibitors and immunostimulators (Figures 5D, E) revealed that the majority of statistically significant molecules were positively correlated with GLI1, including CD160, KDR, CD80, CD28, KLRK1, and HHLAF. Furthermore, examination of immune checkpoint genes (Figures 5F, G) demonstrated a positive correlation between GLI1 and certain immune checkpoint suppressor genes, such as IL4, as well as with over half of the immune checkpoint promoting genes, including CD80, IL1A, CD40LG, TLR4, and IFNA1,2.

Furthermore, by utilizing the immune therapy-related scores of various samples in the TCIA clinical database, we observed significant differences in the response among patients with high and low expression of the GLI1 gene. Specifically, there were significant differences in response for patients who were positive for CTLA-4 treatment and positive for PD-1 treatment (*p* = 1.7e-10), positive for CTLA-4 treatment and negative for PD-1 treatment (*p* = 5.2e-9), negative for CTLA-4 treatment and positive for PD-1 treatment (P = 4e-12), and negative for CTLA-4 treatment and negative for PD-1 treatment (*p* = 2.6e-10) (Figures 5H–K).

### 3.6 GLI1 expression associated functional pathways

**FIGURE 6:**
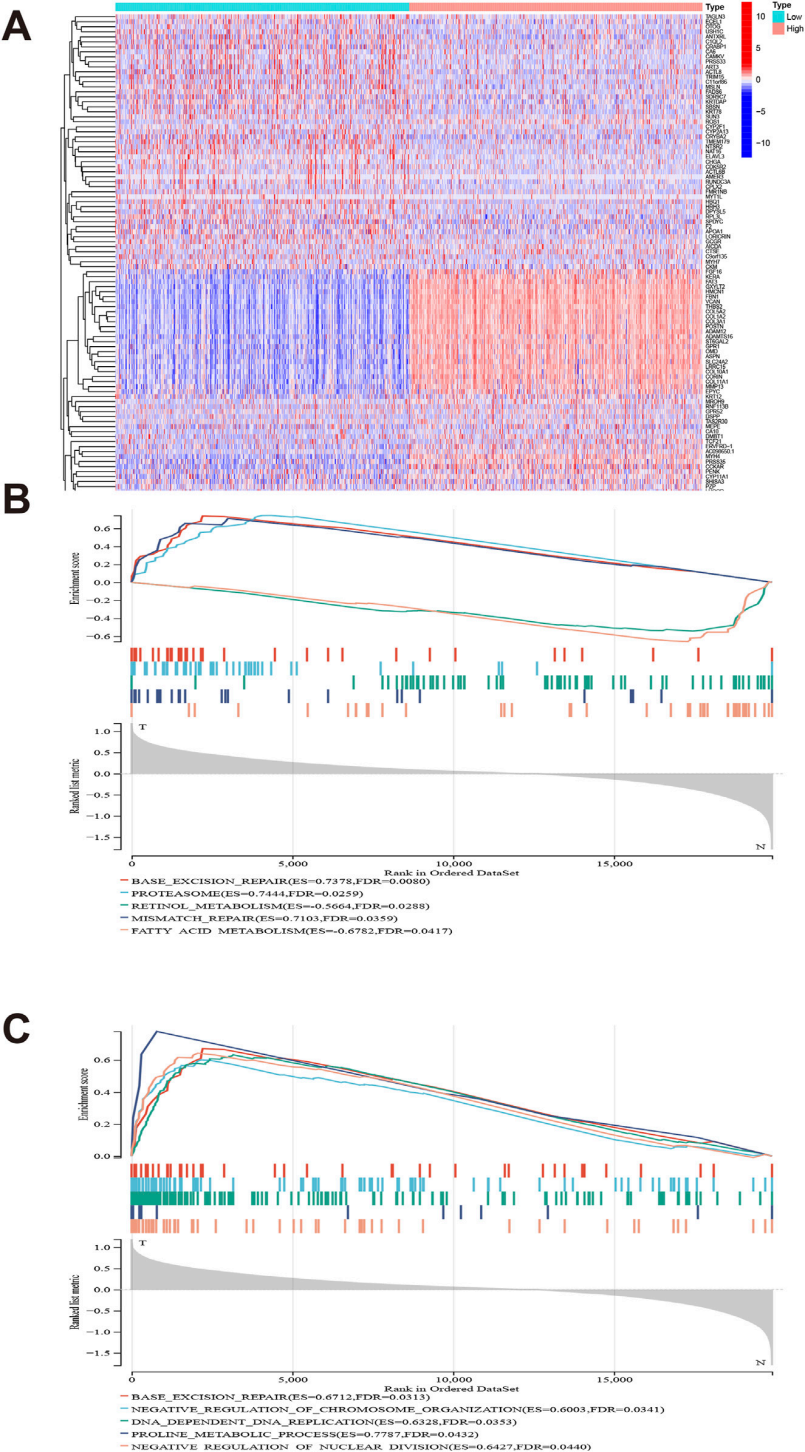
Gene set difference analysis and enrichment analysis. Heat map of high and low differential expression of GLI1 and related genes **(A)**. GLI1 expression is significantly associated with KEGG pathways **(B)** and gene ontology terms **(C)**.

We presents a differential expression analysis of genes associated with GLI1, highlighting significant changes in genes such as KERA, GXYLT2, and COL5A2 relative to GLI1. (Figure 6A). GSEA was employed to investigate the functional pathways significantly associated with GLI1 expression. KEGG pathways, such as proteasome and mismatch repair, exhibited activation, while retinol metabolism showed a negative correlation with high GLI1 expression (Figure 6B). Additionally, several immune-related GO terms were found to be associated with high GLI1 expression, including negative regulation of chromosome organization, DNA-dependent DNA replication, and retinol metabolism (Figure 6C). These findings shed light on the potential mechanism underlying the involvement of GLI1 in immune status.

### 3.7 Prediction of drug sensitivity associated with expression of GLI1 in BRCA

Our study identified notable differences in drug sensitivity profiles among BRCA samples based on GLI1 expression levels.

**FIGURE 7:**
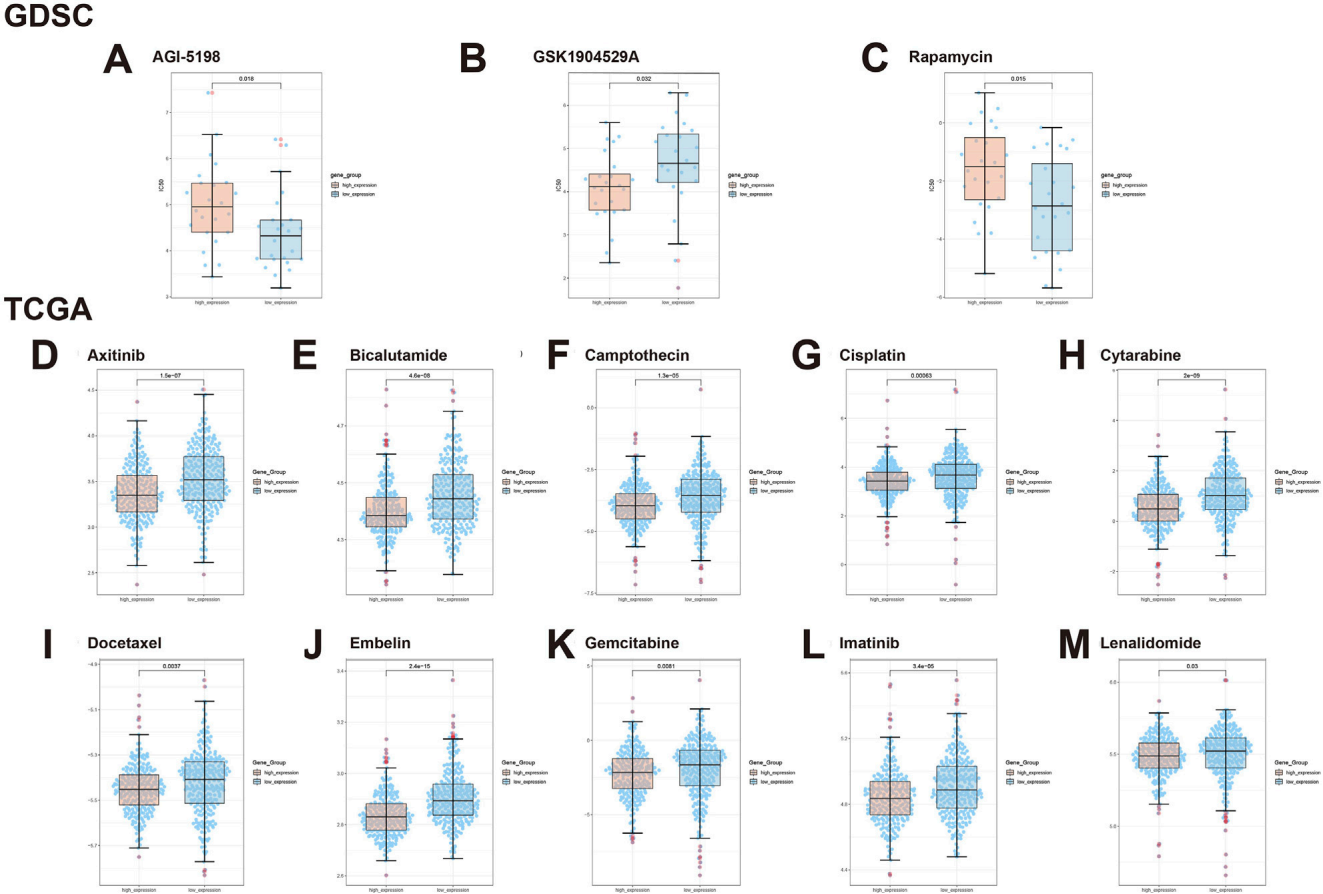
Illustrates the expression of GLI1 in BRCA tumors under the influence of various drugs. **(A)** AGI-5198, **(B)** GSK1904529A, **(C)** Rapamycin, **(D)** Axitinib, **(E)** Bicalutamide, **(F)** Camptothecin, **(G)** Cisplatin, **(H)** Cytarabine, **(I)** Docetaxel, **(J)** Embelin, **(K)** Gemcitabine, **(L)** Imatinib, and **(M)** Lenalidomide.

Based on the GDSC module, we analyzed genomic data of the GLI1 gene and drug sensitivity data in BRCA tumors. We observed significant drug sensitivity of AGI-5198 in the GLI1 low-expression group (*p* = 0.018, Figure 7A), whereas GSK1904529A exhibited significantly reduced sensitivity in the GLI1 low-expression group (*p* = 0.032, Figure 7B). Similarly, Rapamycin showed higher drug sensitivity in the GLI1 low-expression group compared to the high-expression group (*p* = 0.015, Figure 7C).

Using the TCGA module, we further explored the drug sensitivity spectrum of GLI1 in BRCA tumors. Axitinib demonstrated significant resistance in tumors with low GLI1 expression, whereas it exhibited better sensitivity in tumors with high GLI1 expression (*p* = 1.5e-07, Figure 7D). In contrast, Bicalutamide showed higher drug sensitivity in samples with high GLI1 expression (*p* = 4.6e-08, Figure 7E), potentially offering a therapeutic strategy for BRCA tumors. Additionally, Camptothecin (*p* = 1.3e-05, Figure 7F) and Gemcitabine (*p* = 0.0081, Figure 7H) displayed increased sensitivity in BRCA samples with high GLI1 expression. For Cisplatin, we observed a lower half-maximal inhibitory concentration in the high-expression group compared to the low-expression group (*p* = 0.00063, Figure 7G), indicating a correlation between high GLI1 expression and increased drug sensitivity. Furthermore, Docetaxel exhibited significant drug sensitivity in tumors with high GLI1 expression (*p* = 0.0037, Figure 7I), while Embelin showed enhanced sensitivity in samples with high GLI1 expression (*p* = 2.4e-15, Figure 7J). Moreover, Gemcitabine (*p* = 0.0081, Figure 7K) and Imatinib (*p* = 3.4e-05, Figure 7L) demonstrated significantly enhanced drug sensitivity in tumors with high GLI1 expression. Lenalidomide showed higher sensitivity in the high-expression group compared to the low-expression group (*p* = 0.03, Figure 7M).

## 4 Discussion

Breast cancer is a leading cause of mortality among women globally, with its incidence progressively rising. However, the presence of heterogeneity poses challenges to early diagnosis and targeted therapy for breast cancer. Prognosis-associated molecular markers, including ER, PR, HER2, and Mib1/Ki-67, have gained considerable attention due to their demonstrated utility in clinical practice (Yan et al., 2020; Tarighati et al., 2023). Moreover, the emergence of innovative therapies like gene therapy and immunotherapy holds promise for effective treatment (Dastjerd et al., 2022; Yang et al., 2023). Therefore, it is imperative to identify additional prognostic biomarkers and explore new treatment strategies to optimize breast cancer management.

GLI1 has garnered significant attention for its paradoxical and multifunctional properties in various pathologies. It is recognized as a regulator of carcinogenesis and a potential therapeutic target in cancer (Sun et al., 2014; Li et al., 2016). However, the expression and specific roles of GLI1 in breast invasive carcinoma have been scarcely explored. Therefore, our study aims to comprehensively investigate the roles of GLI1 in breast invasive carcinoma.

Initial analysis across cancer types showed that GLI1 expression is downregulated in some (e.g., BLCA, BRCA) but upregulated in others (e.g., CHOL, KIRC), compared to normal tissues. This finding contradicted previous reports that showed elevated GLI1 expression in many cancers (Kubo et al., 2004; Wu and Dai, 2017; Sahu et al., 2022). The disparity in results may be attributed to sample differences and the paradoxical nature of GLI1. The differential expression of GLI1 in diverse cancers underscores its significance and potential as a therapeutic target.

Among the 33 cancer types studied, GLI1 exhibited lower expression in female tumors, including BRCA, CESC, and UCEC. In breast invasive carcinoma, GLI1 expression was significantly higher in normal tissue compared with tumor tissue. Immunohistochemical staining of clinical samples further confirmed the low expression of GLI1 in tumor tissue. Notably, patients with lower GLI1 expression during the first 8 years of illness experienced worse survival outcomes. Further correlation analysis of clinical data revealed differences in GLI1 expression between patients older and younger than 65 years old, as well as among different pathological stages and T stages. These differences held certain guiding significance. Consequently, this indirectly explained why BRCA patients with low GLI1 expression had a poorer survival outcome. Effective prognostic biomarkers play a crucial role in clinical management and treatment decision-making, providing essential information regarding tumor progression and clinical outcomes. Our analyses confirm that GLI1 serves as a prognostic biomarker in breast invasive carcinoma, correlating with pathological stages and survival outcomes in this disease.

Multiple immunotherapies, such as CTLA-4-, PD-1-, and PD-L1-specific immune checkpoint antagonists, have been developed in breast cancer to effectively enhance patient survival. However, currently available immunotherapies for breast cancer have been associated with drug resistance and adverse events (thyroid dysfunction and adrenocortical hypofunction, etc.). The effectiveness of immunotherapy primarily relies on the infiltrating immune cells within the TMIE, which play a crucial role in modulating tumor progression through dynamic and extensive crosstalk with tumor cells (Zhang et al., 2020; Yang et al., 2022). Various molecules have been shown to participate in this intercellular crosstalk (Inaguma et al., 2013; Zhang et al., 2017).

Our study explores the expression pattern of GLI1 in BRCA and lays the foundation for investigating its potential immunoregulatory role, with a focus on correlating GLI1 expression with immune cell infiltration and checkpoint molecules. Additionally, functional hypotheses are proposed based on the observed correlations to further investigate the underlying mechanisms of GLI1 in BRCA immunity.

High expression of GLI1 positively correlated with the abundance of infiltrating immune cells, such as DCs and CD4^+^ T cells, suggesting that GLI1 may partially regulate the recruitment of immune cells in the TMIE of breast invasive carcinoma. Furthermore, we observed a strong correlation between high GLI1 expression and multiple immune checkpoints, immune status-related genes, and chemokine-related genes. Correlation analysis of immunotherapy demonstrated significant differences in efficacy between groups with high or low expression of GLI1 under different immunotherapy regimens, indicating that GLI1 may influence immunotherapy through potential mechanisms.

Based on the observed correlations between GLI1 expression and immune cell infiltration, as well as its co-expression with immune-related genes, we hypothesize that GLI1 plays a pivotal role in modulating the immune microenvironment within BRCA. Specifically, we propose that GLI1 may regulate immune cell infiltration and activation by influencing the expression of chemokines, cytokines, and other immune modulators. Moreover, the association of GLI1 with immune checkpoint molecules, such as CTLA-4 and PD-1, suggests that GLI1 may influence the efficacy of immunotherapy by modulating immune cell exhaustion and activation. To validate these hypotheses, further functional studies, including *in vitro* cell culture experiments and *in vivo* animal models, are warranted to examine the direct effects of GLI1 on immune cell function and BRCA tumor progression. Such experiments would provide crucial insights into the molecular mechanisms underlying the immunoregulatory role of GLI1 in BRCA and potentially guide the development of novel immunotherapeutic strategies targeting GLI1.

In the context of our study, the observed activation of proteasome, inhibition of mismatch repair (MMR), and the negative correlation with retinol metabolism pathways, as well as the differential GO terms, offer important insights into the complex mechanisms underlying GLI1-mediated breast cancer progression. Specifically, the activation of the proteasome pathway highlights GLI1’s potential role in regulating protein homeostasis, which is crucial for maintaining cellular functions and processes that drive cancer progression. By modulating the degradation of GLI1 and other proteins, the proteasome may influence GLI1’s oncogenic activities, as suggested by previous studies (Morrison et al., 2020). This finding underscores the need for further investigation into the specific mechanisms linking proteasome activity and GLI1 regulation in breast cancer. Moreover, the inhibition of MMR pathways observed in our study aligns with previous reports indicating that GLI1 can interfere with MMR, potentially leading to genomic instability and accelerated cancer progression (Inaguma et al., 2013). This finding further emphasizes the oncogenic potential of GLI1 in breast cancer and highlights the importance of understanding its role in maintaining genomic stability. Interestingly, the negative correlation between retinol metabolism and GLI1 high expression suggests a potential regulatory loop between these two pathways. Retinoic acid, a metabolite of retinol, has been shown to regulate GLI1 expression and Hedgehog signaling (Hong et al., 2024), which is frequently dysregulated in breast cancer (An et al., 2021). Our results indicate that this regulatory loop may be disrupted in GLI1-high breast cancer cells, leading to alterations in retinol metabolism and contributing to cancer progression. This finding has important implications for breast cancer therapy, as targeting retinol metabolism or Hedgehog signaling may represent novel therapeutic strategies for GLI1-driven breast cancers.

Due to the preliminary nature of this study, limitations exist in understanding how GLI1 is adapted and regulated by immunotherapy. Further in-depth mechanistic investigations are necessary, such as employing transwell invasion assays and wound healing assays to explore the impact of GLI1 on cellular behavior within the immune microenvironment of invasive breast cancer (Hong et al., 2024; Wang et al., 2023). Additionally, targeting GLI1 as a key gene may facilitate the screening of effective therapeutic agents (An et al., 2021), contributing to the advancement of personalized treatment approaches for breast cancer and promoting relevant clinical trials for combination immunotherapy.

## 5 Conclusion

In summary, this study extensively investigates the expression of GLI1 in BRCA and its immunoregulatory role. The findings provide evidence of low GLI1 expression in BRCA, which may serve as a prognostic indicator for patient survival. We have established a close association between GLI1 and the immune status of BRCA, indicating its potential as a biomarker or specific target for immunotherapy in BRCA. Further research is warranted to elucidate the role of GLI1 in BRCA.

## Data Availability

Publicly available datasets were used in this study. The TCGA gene expressions along with the clinical datasets were downloaded from the TCGA (https://tcga.xenahubs.net), GTEx database (http://commonfund.nih.gov/GTEx/) and TCIA database (https://tcia.at/).
