# Supplementary material for: Glioma-associated oncogene homolog 1 in breast invasive carcinoma: a comprehensive bioinformatic analysis and experimental validation
Source: Front Cell Dev Biol. 2024 Oct 17;12:1478478. doi: 10.3389/fcell.2024.1478478 (PMC11524909; doi:10.3389/fcell.2024.1478478)
Supplement: Supplementary file 1 [file DataSheet1.docx]

**Supplementary Figure 1** Immunohistochemistry results for patient 3-5.

**
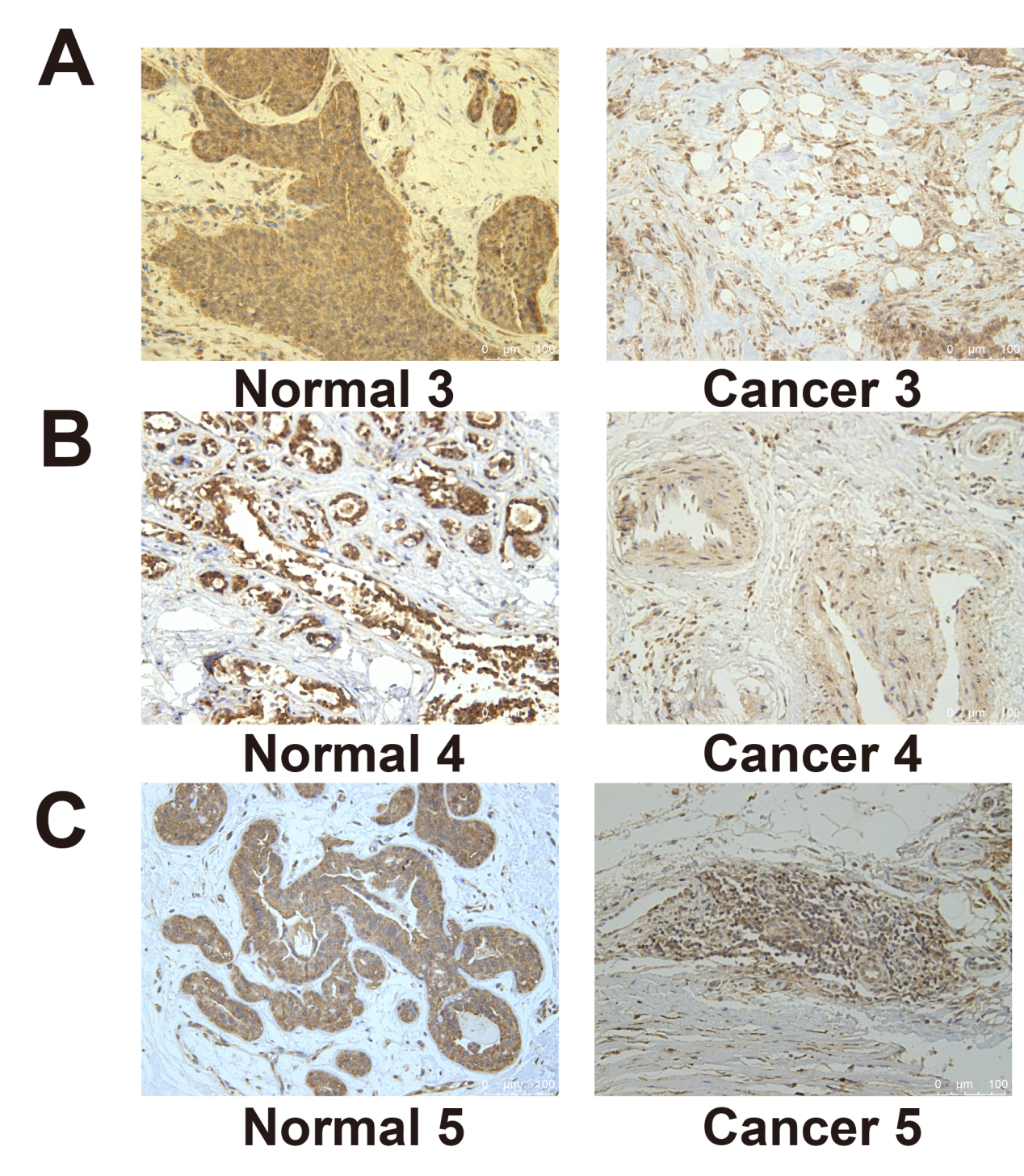
**

**Supplementary Table 1** The abbreviations and sample sizes corresponding to 33 types of tumors

| Abbreviations | Cancer type | Tumor sample | Normal sample |
| --- | --- | --- | --- |
| ACC | Adrenocortical carcinoma | 79 | 0 |
| BLCA | Bladder urothelial carcinoma | 408 | 19 |
| BRCA | Breast invasive carcinoma | 1093 | 112 |
| CESC | Cervical squamous cell carcinoma and endocervical adenocarcinoma | 304 | 3 |
| CHOL | Cholangiocarcinoma | 36 | 9 |
| COAD | Colon adenocarcinoma | 457 | 41 |
| DLBC | Lymphoid neoplasm diffuse large B-cell lymphoma | 48 | 0 |
| ESCA | Esophageal carcinoma | 184 | 11 |
| GBM | Glioblastoma multiforme | 153 | 5 |
| HNSC | Head and neck squamous cell carcinoma | 520 | 44 |
| KICH | Kidney chromophobe | 66 | 25 |
| KIRC | Kidney renal clear cell carcinoma | 533 | 72 |
| KIRP | Kidney renal papillary cell carcinoma | 290 | 32 |
| LAML | Acute myeloid leukemia | 173 | 0 |
| LGG | Brain lower grade glioma | 516 | 0 |
| LIHC | Liver hepatocellular carcinoma | 371 | 50 |
| LUAD | Lung adenocarcinoma | 515 | 59 |
| LUSC | Lung squamous cell carcinoma | 501 | 51 |
| MESO | Mesothelioma | 87 | 0 |
| OV | Ovarian serous cystadenocarcinoma | 303 | 0 |
| PAAD | Pancreatic adenocarcinoma | 178 | 4 |
| PCPG | Pheochromocytoma and paraganglioma | 179 | 3 |
| PRAD | Prostate adenocarcinoma | 497 | 52 |
| READ | Rectum adenocarcinoma | 166 | 10 |
| SARC | Sarcoma | 259 | 0 |
| SKCM | Skin cutaneous melanoma | 103 | 0 |
| STAD | Stomach adenocarcinoma | 415 | 35 |
| TGCT | Testicular germ cell tumors | 150 | 0 |
| THCA | Thyroid carcinoma | 501 | 59 |
| THYM | Thymoma | 120 | 0 |
| UCEC | Uterine corpus endometrial carcinoma | 545 | 35 |
| UCS | Uterine carcinosarcoma | 57 | 0 |
| UVM | Uveal melanoma | 80 | 0 |

**Supplementary Table 2** Detailed diagnostic information for all patients.

| No. | Diagnosis | Age | Gender | WHO | Location |
| --- | --- | --- | --- | --- | --- |
| 1 | IBC-NST | 68 | Female | III | Left |
| 2 | IBC-NST | 61 | Female | III | Left |
| 3 | IBC-NST | 50 | Female | III | Left |
| 4 | IBC-NST | 75 | Female | II | Right |
| 5 | IBC-NST | 69 | Female | II | Right |

Abbreviation:IBC-NST, Invasive Breast Carcinoma of No Special Type.
